# Recurrent microbial keratitis and endogenous site *Staphylococcus aureus* colonisation

**DOI:** 10.1038/s41598-020-75821-z

**Published:** 2020-10-29

**Authors:** Tobi F. Somerville, Jayendra Shankar, Sarah Aldwinckle, Henri Sueke, Timothy Neal, Malcolm J. Horsburgh, Stephen B. Kaye

**Affiliations:** 1 Department of Eye and Vision Sciences, University of Liverpool, 6 West Derby Street, Liverpool, L7 8TX UK; 2 St Paul’s Eye Unit, The Royal Liverpool University Hospital, Liverpool, UK; 3 Department of Microbiology, The Royal Liverpool University Hospital, Liverpool, UK; 4 Department of Infection Biology and Microbiomes, University of Liverpool, Liverpool, UK

**Keywords:** Corneal diseases, Infection

## Abstract

This study investigated *Staphylococcus aureus* carriage in patients with microbial keratitis (MK). 215 patients with MK, 60 healthy controls and 35 patients with rheumatoid arthritis (RA) were included. Corneal scrapes were collected from patients with MK. Conjunctival, nasal and throat swabs were collected from the non-MK groups on a single occasion and from the MK group at presentation and then at 6 and 12 weeks. Samples were processed using conventional diagnostic culture. 68 (31.6%) episodes of clinically suspected MK were classed as recurrent. Patients with recurrent MK had a higher isolation rate of *S. aureus* from their cornea than those with a single episode (p < 0.01) and a higher isolation rate of *S. aureus* from their conjunctiva compared to control participants, 20.6% (14/68) versus 3% (5/60) respectively (p = 0.01). Significantly more patients with recurrent MK (12/68, 17.6%) were found to have *S. aureus* isolated from both their conjunctiva and nose than those with a single episode of MK (7/147, 4.8% p = 0.002) and compared to patients in the control group (3/60, 5.0% p = 0.03). The results indicate that patients with recurrent MK have higher rates of carriage of *S. aureus* suggesting endogenous site colonisation as a possible source of recurrent infection.

## Introduction

Microbial keratitis (MK) is a major cause of loss of vision worldwide with each episode leading to further corneal scarring and visual impairment^1–3^. Treatment involves managing any other associated ocular surface disease involvement, removing any known risk factors such as contact lens wear and aggressive topical antimicrobial therapy. The patient requires close treatment response monitoring, often including hospitalisation followed by frequent outpatient visits. The risk of developing MK and the severity of the disease depend on both the identity of the infecting organism and the condition of the ocular surface^4^. We recently carried out a retrospective case series that included 2418 patients with clinically suspected MK over a 16-year period. Twelve percent of patients were found to have at least two episodes, 3.68% at least three, 1.65% at least four and 0.91% five or more episodes. In patients who had recurrent episodes of MK, *Staphylococcus aureus* was isolated significantly more frequently than other pathogenic bacteria, possibly suggesting an endogenous source for the recurrent nature of *S. aureus* related MK^5^. *S. aureus*, is considered a pathogen in ocular surface infections in contrast to coagulase negative staphylococci (CNS), the predominant isolate cultured from healthy eyes^6,7^.

*S. aureus* is a major human pathogen that can cause wound infections, bacteraemia and sepsis associated with a high mortality rate. The bacterium is a commensal of the moist squamous epithelium of the anterior nares with persistent carriage in 20% of the healthy human population^8^. A higher prevalence of *S. aureus* nasal colonisation has been found in patients with autoimmune diseases such as rheumatoid arthritis (RA) then in the general population^9,10^. Previous studies have also revealed that nasal carriers are at increased risk of acquiring *S. aureus* related infections^8^. Many host and bacterial determinants for nasal carriage are defined but the underlying processes of how carriage is established and maintained are largely undefined^11^.

A key feature of non-ocular *S. aureus* infection is its recurrence, which is seen in approximately 30% of all cases^12,13^. While recurrent or persistent carriage might predispose individuals to recurrent *S. aureus* keratitis, especially those with underlying ocular surface disease this link has yet to be demonstrated. Identification of endogenous host sites such as the conjunctiva, nose and throat are of particular importance for developing interventions to prevent further episodes of MK and subsequent vision loss, particularly since the emergence of methicillin-resistant *Staphylococcus aureus* (MRSA). We therefore, investigated *S. aureus* carriage in the conjunctiva, nose or throat in patients with single episode and recurrent MK in comparison to control groups.

## Results

In total, 215 patients with clinically suspected MK, 60 healthy controls and 35 patients with RA were included. 68 (31.6%) episodes of clinically suspected MK were recurrent within the study period. Baseline characteristics demographics and predisposing risk factors of the 215 patients with clinically suspected MK are presented in Table 1. Patients with recurrent MK had higher rates of risk factors such as RA, Sjogren’s syndrome, ectodermal dysplasia and ocular surface disease and were more likely to be on systemic immunosuppression (Table 1). Conversely, patients with single episode MK had higher rates of exogenous risk factors such as contact lens wear and corneal trauma (Table 1).

**Table 1:** Baseline demographics and predisposing risk factors of patients with clinically suspected MK.

	Single episode MK (n = 147)	Recurrent MK (n = 68)	P-value
Age (median, IQR)	48 years (30.5)	54 years (19.3)	0.21
Males	79 (53.7%)	30 (44.1%)	0.19
**Systemic risk factors**			
Diabetes mellitus	10 (6.8%)	6 (8.8%)	0.60
Rheumatoid arthritis	6 (4.1%)	16 (23.5%)	< 0.01*
Sjogren’s syndrome	1 (0.7%)	6 (8.8%)	0.004*
Ectodermal dysplasia	0	4 (5.9%)	0.009*
Systemic immunosuppression	5 (3.4%)	13 (19.1%)	< 0.001*
**Ocular risk factors**			
Contact lens wear	61 (42.5%)	7 (10.3%)	< 0.001*
Ocular surface disease	64 (43.5%)	46 (67.6%)	0.001*
Corneal trauma	20 (13.6%)	2 (2.9%)	0.02
Topical steroid prior to presentation	12 (8.2%)	12 (17.6%)	0.06

*MK* microbial keratitis.

*Significant after Bonferroni correction.

Corneal scrapes were obtained in 204/215 patients. The corneal scrape isolation rate was 25.5% (52/204). A topical antimicrobial had been used immediately prior to presentation in 83 (26.3%) of clinically suspected MK patients. The corneal scrape isolation rate was no different for patients who had or had not received an antimicrobial (21/80 (26.3%) and 31/124 (25.0%) (p = 0.84), respectively. The microbial isolates are shown in Table 2. Although there was no significant difference in the corneal scrape isolation rate between those that had recurrent MK and those who presented with a single episode (p = 0.53), recurrent MK patients were found to have significantly higher corneal scrape isolations of *S. aureus* than those MK patients presenting with a single episode, that is, 5/13 (38.5%) of positive scrapes in the recurrent MK group versus 2/39 (5.1%) positive scrapes in the single episode MK group (p = 0.001). Higher levels of Gram-negative bacilli, particularly *Pseudomonas* species were found in the single episode MK group, 12.9% versus 4.4% respectively (p = 0.055).

**Table 2:** Isolates from cases of presumed microbial keratitis grouped according to patients with recurrent episodes and those presenting with only a single episode.

	Single episode MK (n = 146)	Recurrent MK (n = 58)
**Total number of positive samples**	39* (26.7%)	13* (22.4%)
**Gram-positive cocci**	19 (13.0%)	10 (17.2%)
*S. aureus*	2 (1.4%)	5 (8.6%)
CNS	11 (7.5%)	3 (5.2%)
*S. pneumoniae*	4 (2.7%)	2 (3.4%)
*Alpha-haemolytic streptococci*	2 (1.4%)	1 (1.7%)
**Gram-positive bacilli**	1 (0.7%)	1 (1.7%)
*Bacillus* sp*.*	0	1 (1.7%)
Diptheroids	1 (0.7%)	0
**Gram-negative bacilli**	19 (13.0%)	3 (5.2%)
*Pseudomonas aeruginosa*	9 (6.1%)	1 (1.7%)
*Moraxella* sp.	4 (2.7%)	0
*Kingella*	1 (0.7%)	0
*Proteus*	0	1 (1.7%)
*Citrobacter koseri*	1 (0.7%)	0
*Acinetobacter lwoffii*	1 (0.7%)	0
*Serratia marcescens*	1 (0.7%)	0
*E.coli*	1 (0.7%)	1 (1.7%)
Other Enterobacteriaceae sp.	1 (0.7%)	0
**Protozoa**	1 (0.7%)	0
A*canthamoeba* sp.	1 (0.7%)	0
**Fungi**	1 (0.7%)	0
*Aspergillus* sp.	1 (0.7%)	0

*The total number of isolates do not add up to 39 and 13 respectively because one recurrent patient had mixed infection with *S. aureus*, *E.coli* and *S. pneumoniae* and two single episode patients had mixed infection with Enterobacteriaeceae and Alpha-Haemolytic Streptococci, and *Pseudomonas aeruginosa* and *Aspergillus* sp, respectively.

*MK* microbial keratitis, *CNS* coagulase-negative Staphylococci.

### Conjunctival, nasal and throat *S. aureus* carriage

Patients with recurrent MK were found to have a significantly higher isolation rate of *S. aureus* from their conjunctiva on presentation compared to control participants, 20.6% (14/68) versus 3% (5/60) respectively (p = 0.01), Table 3. No difference was found between the isolation rates of *S. aureus* from the conjunctiva of patients with non-recurrent MK on presentation compared to control participants (p = 0.77). Isolation rates of *S. aureus* from the nose and throat were not found to be significantly higher in either the single or recurrent MK patient groups compared to controls, Table 3.

**Table 3:** Numbers of individuals in each group who had *S. aureus* isolated from their conjunctiva, nose and throat on presentation.

	Control (n = 60)	Single episode MK (n = 147)		Recurrent MK (n = 68)		MK with positive scrape (n = 52)	
Conjunctiva	3 (5.0%)	9 (6.1%)	P = 1.00	**14 (20.6%)**	**P = 0.02**	6 (11.5%)	P = 0.30
Nose	18 (30.0%)	47 (32.0%)	P = 0.66	29 (42.6%)	P = 0.12	17 (32.7%)	P = 0.66
Throat	4 (6.8%)	19 (12.9%)	P = 0.33	10 (14.7%)	P = 0.26	9 (17.3%)	P = 0.14

P-values represent comparisons of each group to the control group.

*MK* microbial keratitis.

Significantly more patients in the recurrent MK group were found to have *S. aureus* isolated from both their conjunctiva and nose than patients in the single episode MK group, 12/68 (17.6%) versus 7/147 (4.8%) (p = 0.002) respectively and compared to patients in the control group (3/60, 5.0% p = 0.03).

Conjunctival, nose and throat carriage of *S. aureus* was seen to significantly decline over the 3 visits only in the recurrent, clinically suspected bacterial keratitis group (Table 4). Of the 7 MK patients who had *S. aureus* isolated from their corneal scrape, 2 participants consistently had *S. aureus* isolated from their conjunctiva at all three visits.

**Table 4:** Microbial keratitis participants overall *S. aureus* carriage (total *S. aureus* conjunctival, nasal and throat carriage) at presentation (visit 1), 6 weeks post presentation (visit 2) and 12 weeks post presentation (visit 3).

	Single episode MK	Recurrent MK	MK positive scrape episodes	*S. aureus* MK episodes
Visit 1	75/441 (17.0%)	53/204 (26.0%)	32/156 (20.5%)	6/21 (28.6%)
Visit 2	47/303 (15.5%)	26/168 (15.5%)	26/117 (22.2%)	5/18 (27.8%)
Visit 3	40/237 (16.9%)	26/141 (18.4%)	17/96 (17.7%)	4/12 (33.3%)
P value	0.85	0.04	0.72	0.94

Values represent the total number of swabs obtained (nasal swabs were classified as one value and counted as positive regardless if one or both nares were positive). P-values calculated using repeated measures logistic regression (Wald Chi-Square) and represent change over time in *S. aureus* colonization prevalence for each of the four subject groups.

*MK* microbial keratitis.

There was no difference in the isolation rate of *S. aureus* from the conjunctiva, nares and throat in patients who had or had not received an antimicrobial: 8/83 (9.6%) and 15/132 (11.4%) (p = 0.69); 29/83 (34.9%) and 47/132 (35.6%) (p = 0.92) and 11/83 (13.3%) and 18/132 (13.6%) (p = 0.94)), respectively.

### Comparison between patients with RA and healthy controls

Twenty-nine of the 35 patients with RA were on systemic immunotherapy which included monotherapy or dual therapy with methotrexate (21), etanercept (3) and prednisolone (7). There was no statistically significant difference in the isolation rates of *S. aureus* from the conjunctiva, nose and throat between healthy controls and patients with RA (whether or not on systemic immunotherapy), Table 5. There was a non-significant trend (p = 0.06) for patients with RA on systemic immunotherapy to have more *S. aureus* nasal carriage then those patients with RA not on systemic immunotherapy, 13/29 (44.8%) versus 0 respectively, (Table 5).

**Table 5:** Numbers of individuals in the healthy control group versus patients with RA on systemic immunotherapy and patients with RA not on systemic immunotherapy who had S. aureus isolated from their conjunctiva, nose and throat.

	Healthy controls (n = 60)	Patients with RA on systemic immunotherapy (n = 29)		Patients with RA not on systemic immunotherapy (n = 6)		Difference between RA groups
Conjunctiva	3 (5.0%)	0	P = 0.55^a^	0	P = 1.00^b^	–
Nose	18 (30.0%)	13 (44.8%)	P = 0.17^a^	0	P = 0.18^b^	P = 0.06
Throat	4 (6.8%)	3 (10.3%)	P = 0.68^a^	0	P = 1.00^b^	P = 1.00

^a^P-values represent comparison between patients with RA on systemic immunotherapy and healthy controls.

^b^P-values represent comparison between patients with RA not on systemic immunotherapy and healthy controls.

*RA* rheumatoid arthritis.

## Discussion

Recurrent MK is common but little attention has been paid to potential reservoirs of infection and optimal management. This study is the first prospective study to establish a potential source of recurrent MK by comparing multiple endogenous site testing between MK patients and healthy controls over time. Similar to a previous study we found *S. aureus* to be the most frequently associated organism in recurrent MK^5^. We found that *S. aureus* was present in the conjunctiva significantly more frequently in patients who had recurrent MK compared to healthy controls or patients with RA. In addition, patients who had recurrent episodes of MK were found to have significantly more concurrent *S. aureus* conjunctiva and nasal carriage than patients who had single episode and also in comparison to patients in the healthy control group. This would suggest that the conjunctiva and nose and possibly other unidentified sites such as the lid margin may be endogenous sources of *S. aureus* in patients with recurrent MK.

The pathogenicity of *S. aureus* is mediated by a multitude of factors including bacterial surface adhesins, immune evasive proteins and toxins including alpha-, beta- and gamma-toxin plus several leukocidins that mediate tissue damage and contribute towards the induction of the inflammatory response^15–18^. Modern molecular diagnostic tools such as PCR and genome sequencing have provided a better understanding of the composition of the ocular surface microbiome and how this changes with conditions such as: contact lens wear^19^, antibiotic exposure^20^, infectious states^21^, atopic and dry eye disease^22–25^ as well as the effects of age and gender^26^. Presence of *S. aureus* on the ocular surface has been associated with dry eye and atopia^22–25^ which are both known risk factors for MK. In this study, we have demonstrated that patients with recurrent MK are more likely to have endogenous risk factors such as RA, Sjogren’s syndrome, ectodermal dyplasia, ocular surface disease and are more likely to be on systemic immunosuppression. These conditions may predispose to an increased risk of ocular surface *S. aureus* colonisation^27^.

Patients with RA with corneal ulceration have an increased risk of mortality and eye morbidity from infection and a much poorer prognosis than patients with RA without corneal ulceration, and infection is a significant risk factor^14^. Although we did not find a significant difference in *S. aureus* nasal carriage in patients with RA between those with or without systemic immunotherapy, this may reflect that only 6 of the 35 patients with RA were not on immunotherapy. This trend would be consistent with Goodman et al., who found that patients with RA on biological drugs, such as etanercept in our study group, had a higher prevalence of *S. aureus* colonisation (37%) compared to those with RA on disease-modifying anti-rheumatic drugs (DMARDs) alone (24%) or control subjects affected by osteoarthritis (20%)^27^.

We identified no significant difference in *S. aureus* nasal and throat colonisation between patients with a single episode and recurrent keratitis and healthy controls, although it is possible that our sample size was too small to detect a difference between these groups for *S. aureus* nasal and throat carriage. In particular, one of the limitations of this study was the low isolation rate from corneal scrapes and it is therefore possible that other bacteria from the conjunctiva and nose were associated with MK.

In this study we identified an increased isolation of *S. aureus* from endogenous sites in patients with recurrent MK compared to patients with a single episode of MK and this highlights the importance of collecting samples from the conjunctiva and nose in patients with recurrent keratitis. With the use of modern molecular diagnostic tools, it would be useful to examine whether the strains of *S. aureus* obtained from endogenous sites such as the conjunctiva, nose and skin are the same as those obtained from the corneal ulcer and whether patients with recurrent MK are persistently infected with the same strain of *S. aureus* or have intermittent carriage of different strains. A study in which volunteers were artificially inoculated nasally with a mixture of *S. aureus* strains showed that non carriers quickly eliminated the inoculated *S. aureus* strains whereas most persistent carriers selected their original resident *S. aureus* strain from the inoculation mixture^28^. The authors concluded that host characteristics substantially determine the *S. aureus* nasal carrier state and it this therefore likely that a similar process occurs in the conjunctiva. Collecting isolates from patients with recurrent MK and resolving their relatedness (comparative genomics) between episodes would enable the development of decolonisation strategies to reduce the risk of recurrent infection related visual loss.

## Methods

All included patients provided informed consent. The study received prospective ethical approval from the Northwest NHS Research Ethics Committee and was conducted according to the ethical standards set out in the 1964 Declaration of Helsinki, as revised in 2000.

Consecutive patients presenting with suspected MK between January 2011 and November 2012 at The Royal Liverpool University Hospital were recruited and followed up at 6 and 12 weeks. Demographical and clinical information were collected through patient interview and medical notes review. Corneal scrapes were collected from the corneal ulcer at presentation. At presentation and at each subsequent visit conjunctival swabs from each eye, a nasal swab from each nostril and a throat swab were collected. All patients received a topical fluoroquinolone as initial treatment.

A recurrent episode was defined as a prior or further episode of clinically suspected MK occurring in the same patient (in either eye) either more than 3 months before or after the index episode or within 3 months of the index episode if there was documentation that the corneal ulcer had healed in the interim.

In addition to patients with MK, patients with no previous history of MK and current eye drop use, and patients with no previous history of MK and eye drop use but a history of RA, were recruited to a healthy control group and a RA group. These patients were recruited from patients attending for routine cataract surgery and from those attending a RA clinic. Conjunctival, nasal and throat swabs were collected from both the healthy and RA group on a single occasion.

### Sampling and microbial processing

Corneal samples were taken by scraping the edges of the ulcer with a number 11 blade (Swann Morton, Sheffield, UK) whilst wearing sterile gloves, as previously described^29,30^. The blade was placed into enrichment culture broth (brain heart infusion, BHI) and transported to the microbiology laboratory at room temperature^29^. Conventional diagnostic culture was carried out as previously described^30^. Briefly, BHI bottles were vortexed for 5–10 s and 10 μl aliquots inoculated onto blood, chocolate, fastidious anaerobic and Sabouraud’s dextrose agar plates and Robertson’s cooked meat enrichment broth (Oxoid, Basingstoke, UK). For *Acanthamoeba* spp. culture, 20 μl of BHI was inoculated onto a non-nutrient agar (Oxoid) seeded with *Escherichia coli.* All plates were incubated overnight at 37 °C under both aerobic and enriched carbon dioxide (5%) atmospheric conditions. Agar plates and a 24 h subculture of the BHI broth in enrichment medium were examined for evidence of bacterial growth after 24 and 48 h incubation. All isolates were identified using matrix-assisted laser desorption ionization-time-of-flight mass spectrometry (Bruker, Bremen, Germany). Acanthamoeba plates were examined daily for 7 days for the characteristic appearances of acanthamoeba trophozoites and cysts^30^.

Conjunctival, nasal and oral swabs were inoculated onto blood, chocolate, fastidious anaerobic and Sabouraud’s dextrose agar plates (Oxoid, Basingstoke, UK) and processed as above.

### Statistical analysis

All data collected in the study were entered into an electronic database via Microsoft Excel 2016 and analysed using SPSS (version 26). The independent T-test was used to assess differences for normally distributed continuous variables and the χ^2^ was used as indicated for the analysis of categorical variables where the number of observations was 5 or larger. For small-sized samples (fewer than five observations), Fisher’s exact test was used. Repeated measures logistic regression was used to analyse the difference between swab results over the 3 visits.

## Data Availability

The datasets generated during and/or analysed during the current study are available from the corresponding author on reasonable request.
